# Safety and *in vivo* Expression of a GNE-Transgene: A Novel Treatment Approach for Hereditary Inclusion Body Myopathy-2

**DOI:** 10.4137/grsb.s2210

**Published:** 2009-05-08

**Authors:** Anagha P. Phadke, Chris Jay, Salina J. Chen, Courtney Haddock, Zhaohui Wang, Yang Yu, Derek Nemunaitis, Gregory Nemunaitis, Nancy S. Templeton, Neil Senzer, Phillip B. Maples, Alex W. Tong, John Nemunaitis

**Affiliations:** 1 Gradalis, Inc., Dallas, TX; 2 MetroHealth Medical Center, Cleveland, OH; 3 Baylor College of Medicine, Houston, TX; 4 Mary Crowley Cancer Research Centers; 5 Texas Oncology PA; 6 Baylor Sammons Cancer Center, Dallas, TX

**Keywords:** muscular dystrophy, recombinant GNE, safety, in vivo, hereditary inclusion body myopathy, gene therapy

## Abstract

Hereditary inclusion body myopathy-2 (HIBM2) is an adult-onset, muscular disease caused by mutations in the GNE gene. HIBM2-associated GNE mutations causing hyposialyation have been proposed to contribute to reduced muscle function in patients with HIBM2, though the exact cause of this disease is unknown. In the current studies we examined pre-clinical *in vivo* toxicity, and expression of the plasmid-based, CMV driven wild-type GNE plasmid vector. The plasmid vector was injected intramuscularly (IM) or systemically (IV) into BALB/c mice, following encapsulation in a cationic liposome (DOTAP:Cholesterol). Single IM injections of the GNE-lipoplex at 40 μg did not produce overt toxicity or deaths, indicating that the no observable adverse effect level (NOAEL) dose for IM injection was ≥40 μg. Single intravenous (IV) infusion of GNE-lipoplex was lethal in 33% of animals at 100 μg dose, with a small proportion of animals in the 40 μg cohort demonstrating transient toxicity. Thus the NOAEL dose by the IV route was greater than 10 μg and less than or equal to 40 μg. Real-time RT-qPCR analysis demonstrated recombinant human GNE mRNA expression in 100% of muscle tissues that received IM injection of 40 μg GNE-lipoplex, at 2 weeks. These results indicate that GNE-lipoplex gene transfer is safe and can produce durable transgene expression in treated muscles. Our findings support future exploration of the clinical efficacy of GNE-lipoplex for experimental gene therapy of HIBM2.

## Introduction

Hereditary inclusion body myopathy-2 (HIBM2) or distal myopathy with rimmed vacuoles (DMRV) is an autosomal, recessive, muscular disorder that is caused by mutations in the GNE gene.^1^–^3^ It is an early adult-onset disease characterized by slowly progressive distal and proximal muscle weakness and atrophy. There is preferential weakness of the tibialis anterior muscles with relative sparing of quadriceps. Histologically, it is characterized by cytoplasmic rimmed vacuoles in atrophic fibers and cytoplasmic or nuclear filamentous inclusions composed of tubular filaments.^4^

HIBM2 has been attributed to a variety of homozygous or compound heterozygous missense mutations in the GNE gene, spanning the entire enzyme including either the epimerase or kinase domains or both. The mutation spectrum is varied, with over 40 mutations found across the whole coding region of the GNE gene.^5^ Cases of HIBM2 confirmed by genetic testing have been found all over the world.^6^–^8^

The GNE gene encodes the bifunctional enzyme uridine diphospho-N-acetylglucosamine (UDP-GlcNAc) 2 epimerase/N acetyl-mannosamine (ManNAc) kinase (GNE/MNK).^9^,^10^ This enzyme is ubiquitously expressed and catalyzes the first 2 steps of the sialic acid biosynthesis pathway.^11^ HIBM2-associated GNE mutations have been shown to result in reduced activity of both GNE and MNK which is responsible for reduced sialic acid production.^12^,^13^ Sialyation of skeletal muscle glycoproteins is necessary for proper folding, stabilization, and function of these proteins.^14^–^16^ GNE mutations resulting in hyposialyation of muscle glycoprotein has been proposed to contribute to myofibrillar degeneration and reduced muscle function in patients with HIBM2, although yet undefined factors may also contribute to the pathology of HIBM2.^2^,^14^,^16^–^18^

Since reduction in sialic acid due to the GNE mutation is considered to be one of the causal factors in the development of HIBM2, we sought to reconstitute wild-type (wt) GNE expression using a gene therapy approach. We constructed a GNE-wt-DNA vector using human GNE cDNA and pUMVC3 expression vector. Our earlier *in vitro* findings indicated that this vector produced high levels of recombinant GNE protein in transfected CHO-Lec3 (GNE deficient cell line) cells that produced low levels of sialic acid.^19^ To characterize its *in vivo* activity, the GNE expression vector was complexed with a cationic liposome (composed of 1,2-Dioleoyl-3-Trimethylammonium-Propane (DOTAP) and cholesterol) to form a 300–500 nm lipoplex (GNE-lipoplex). Complexing DNA with liposomes has been demonstrated to extend the life span of circulating DNA. Prior studies have shown that naked plasmid DNA rapidly degrades *in viv*o, but lipoplexes could be detected hours after injections.^20^

Toxicity studies were performed in mice to delineate preclinical parameters applicable for the design of clinical trials with the GNE-lipoplex. Additionally, we assessed GNE expression via real time RT-qPCR analysis following both intramuscular (IM) and intravenous (IV) delivery of GNE-lipoplex.

## Materials and Methods

### Study animals

Male and female BALB/c mice (9–11 weeks) were purchased from Harlan Sprague Dawley (Indianapolis, Indiana) and housed in an animal facility approved by the Institutional Animal Care and Use Committee at Baylor Research Institute (Dallas, Texas). The animals were grouped into different cohorts for either a single IM or IV injection as outlined in Table 1. Studies I and III were performed to assess the toxicity profile of GNE-lipoplex and examine recombinant human GNE (rGNE) mRNA in various mice tissues. Study II was performed to examine temporal expression of rGNE mRNA in mouse muscle tissue. For Studies I and III mice were observed for 2 weeks at which time surviving mice were sacrificed for gross examination and collection of various tissues for rGNE mRNA expression and histopathology analysis.

### GNE cloning

The parental vector containing wild type human GNE cDNA was provided by Daniel Darvish (HIBM2 Research Group; Encino, CA). The destination vector, pUMVC3, was purchased from Aldevron (Fargo, ND). Wild type GNE was cloned from the parent vector into pUMVC3 via Eco RI restriction digest, gel purification, and T4 ligation. All pUMVC3-GNE clones were sequenced by Seqwright (Houston, TX) in both orientations to confirm the vector identity. Positive pUMVC3-GNE clones were shipped to the Waisman Clinical BioManufacturing Facility, University of Wisconsin-Madison for master cell bank and large scale GMP production.

### Preparation of lipoplexes

Stock 5x DOTAP:Cholesterol liposomes were purchased from GeneExcel (Houston, TX) and diluted to 2x in D5W. Plasmid DNA was diluted in D5W to a concentration of 1mg/ml. An equal volume of diluted plasmid DNA was mixed with the diluted DOTAP:Cholesterol to form the GNE-lipoplex (0.5 mg/ml). For studies I and III, GNE-lipoplex was diluted in D5W to a final concentration of 0.5, 0.2, 0.125, and 0.05 mg/ml so that the animals were injected with an equal volume of material. Empty liposomes were prepared by diluting stock DOTAP:Cholesterol in D5W to a final concentration of 1x. Prepared GNE-lipoplex was stored in glass vials with argon gas at 2–8 °C, until used. The GNE-lipoplex had an average size of 400 nm and a zeta potential of +55 mv. Animals were injected with GNE-lipoplexes within 2 weeks of lipoplex formulation. (After three months, stability studies on remaining GNE-lipoplexes demonstrated no significant change in lipoplex structure.) For study II, the vials containing GNE-lipoplex were warmed to room temperature and mixed with 20% (v/v) 0.2 um filtered India ink, prior to injection. The final injected test article contained 80% GNE-lipoplex and 20% dye.

### Animal injections

Glass vials containing GNE-lipoplex were brought to room temperature and loaded into 1 ml syringes using a 21-gauge needle to reduce disruption of the complexes. The needle was changed to a 30-gauge needle prior to injecting the animals. For Study I, mice were administered a single IM injection of 0 μg, 10 μg, or 40 μg GNE-lipoplex in a total volume of 80 μl. Mice were lightly anesthetized using isoflurane inhalation and injected in the right quadriceps muscle twice, each injection consisting of 40 μl GNE-lipoplex. This precaution was taken to prevent injury to the muscle tissue. For Study II, India ink was filtered though a 0.2 μm filter and mixed (20% v/v) with GNE-lipoplex prior to loading the syringes so that the injected material could be tracked at the time of necropsy. The IM injections were performed as mentioned earlier, for Study I. In Study III, mice were administered 10 μg, 40 μg, or 100 μg of GNE-lipoplex as a single IV injection in a total volume of 200 μl. All three studies included mice that were injected with “empty” liposomes without DNA (vehicle control), with Study I also containing uninjected control mice (Table 1).

### Toxicology assessments

Mice were monitored daily for adverse events and for evidence of overt toxicity. Mice were weighed prior to treatment and once a week for 2 weeks after treatment (studies I and III). On day 14 post-injection, surviving mice in Studies I and III were sacrificed by cervical dislocation following isoflurane vapor inhalation. Necropsy was performed to assess pathological changes. Blood and sera were shipped to SRI laboratories (Mountain View, CA) for evaluation of complete blood counts and blood chemistry profile. The following parameters were considered for toxicology analysis: alanine aminotransferase (ALT), aspartate aminotransferase (AST), creatine kinase, blood urea nitrogen, total bilirubin, albumin, alkaline phosphatase, WBC, RBC, and platelet counts.

The various tissues collected at necropsy, including left uninjected muscle, right injected muscle (only for study I), kidney, spleen, liver, and lungs (Studies I and III), were weighed and divided into 2 pieces each. One tissue piece was snap frozen in liquid nitrogen for molecular analysis. The 2nd tissue piece was embedded in a mold containing optimum cutting temperature (OCT) mounting medium and snap-frozen in liquid nitrogen. The frozen tissue blocks were sectioned at 4 μm thickness and stained with hematoxylin and eosin. The stained slides were shipped to IDEXX Laboratories (West Sacramento, CA) for histopathology analysis by a certified veterinary pathologist. For Study II, spleen, injected, and uninjected muscles were collected from the mice at 24 hr, 48 hr, 72 hr, week 1 and 2 post-injection. These tissues were processed to quantify expression of rGNE mRNA via real-time RT-qPCR.

### RT-qPCR analysis

Total RNA was isolated from the mouse samples by tissue homogenization with the Fast-Prep 24 (MP Biomedical, Solon, OH) and total RNA extracted using the Corbett X-tractor robot (San Francisco, CA) along with the Macherey-Nagel (Bethlehem, PA) NucleoSpin-8 RNA kit. One microgram of total RNA was used as starting material for cDNA synthesis using oligo dT primers and the BioRad (Hercules, CA) iScript Select kit. GNE specific primers were designed so that only the plasmid (human GNE) transcript would be amplified without cross reacting with the endogenous (mouse GNE) mRNA.

### Statistical analysis

Group means/average and standard deviations were calculated for body weights, hematology, and blood chemistry assays. Student’s t-test (p < 0.05) was used to analyze statistical differences among cohorts.

## Results

### No observable adverse effect level (NOAEL) determination for a single IM injection

In order to establish safety of a localized injection of GNE-lipoplex, a single 10 μg or 40 μg dose was administered IM into normal, immunocompetent BALB/c mice. Mice administered with either 10 μg or 40 μg GNE-lipoplex survived without demonstrating overt signs of toxicity for the observation period of 2 weeks (Table 2). Treatment induced weight loss was not evident in injected mice when compared to their baseline weights (mean weight of female mice in grams; pre-treatment: 17.9 ± 1.1, week 1 post treatment: 18.4 ± 1.1, week 2 post-treatment: 19 ± 1.1; mean weight of male mice in grams; pre-treatment: 25.9 ± 1.3, week 1 post treatment: 26.5 ± 1.4, week 2 post-treatment: 26 ± 2.8).

There were no significant differences in the various hematology parameters for the 3 injected cohorts at the end of 2 weeks after exclusion of clotted specimens from the various cohorts (Table 2). Blood chemistry analyses, showed significantly elevated AST and creatine kinase values in mice that received the “empty” liposomes injection (mice that received 0 μg DNA in liposomes) (AST: 305 ± 65 U/L; creatine kinase: 2110 ± 571 U/L; n = 6) as compared with the uninjected cohort (AST: 172 ± 42 U/L; creatine kinase: 1052 ± 290 U/L; n = 6). This suggested toxicity to the liver and muscles of mice in this cohort due to the empty liposomes. The average AST and creatine kinase levels in the 10 μg cohort appeared elevated when compared to the uninjected control group, but these values were not statistically significant due to the high standard deviation as a result of 2 outliers in the 10 μg group (p = 0.2 for AST and creatine kinase assay). AST and creatine kinase levels were not significantly elevated in cohorts that received 40 μg GNE-lipoplex (p = 0.5 and p = 0.8 respectively) (Table 2) suggesting that the elevated values observed for the 10 μg cohort were not due to GNE-lipoplex.

Although the albumin levels were altered in the cohort injected with 10 μg (3.4 ± 0.1 g/dL) as compared with the uninjected cohort (3.1 ± 0.2 g/dL), these values were within the normal range for the assay (Table 2). There were no significant alterations in ALT, total bilirubin, alkaline phosphatase and blood urea nitrogen levels among the various cohorts (Table 2).

None of the organs (liver, lung, spleen, kidney, or muscles) demonstrated any gross pathological changes at necropsy. Muscle inflammation or degeneration was observed in 4 mice in the cohort injected with liposome only, and in 1 mouse that was uninjected. Myodegeneration with or without myositis was observed in 1 mouse in the cohort injected with 10 μg, and 6 mice in the cohort injected with 40 μg GNE-lipoplex. Overall, histopathological analysis did not demonstrate any remarkable findings that could be attributed to GNE-lipoplex treatment. The observed myodegeneration and associated tissue mineralization with myositis (4 mice) or without myositis (8 mice) was probably related to injection site trauma among 36 IM injected mice. These findings indicate that the no observed adverse effect level (NOAEL) dose for a single IM injection was ≥40 μg GNE-lipoplex.

### Recombinant human GNE (rGNE) mRNA expression analysis after IM injection

**Figure 1:**
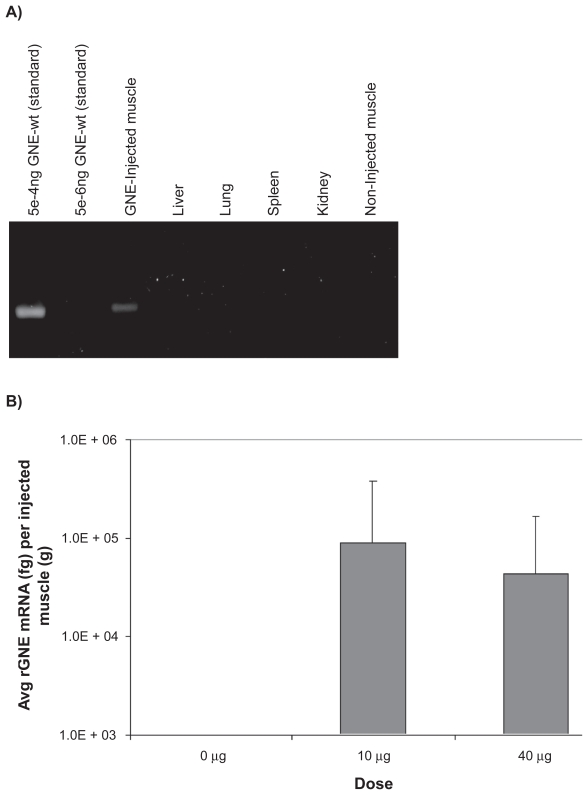
Recombinant human GNE mRNA expression post intramuscular injection. Mice were administered a single IM injection of 0 μg, 10 μg, or 40 μg GNE-lipoplex. At the end of 2 weeks, tissues (non-injected muscle, injected muscle, kidney, spleen, liver, and lungs) were collected. Total RNA was extracted and real-time qPCR analysis was performed. **A**) rGNE mRNA expression and distribution. Plasmid DNA (GNE-wt) was diluted and used as a template to demonstrate the sensitivity of the assay. A representative gel picture illustrates rGNE was only detected in injected muscles from study I. **B**) Mean (± SD) rGNE mRNA expression in injected muscles (n = 12).

Human rGNE mRNA expression in the injected, necropsied muscle tissue was determined by RT-qPCR analysis, using total RNA (300–2500ng/μl) extracted from tissues. An equal amount of total RNA (1 μg) was converted to cDNA via reverse transcription. rGNE mRNA expression was detected in 50% (6/12) of mice at the 10 μg dose in the injected muscle, and in 83% (10/12) of mice at the 40 μg dose. Mean rGNE mRNA expression in the injected muscle was 8.8E + 04 fg at the 10 μg dose and 4.3E + 04 fg at the 40 μg dose (Fig. 1b). There was no detectable rGNE mRNA expression in animals that received the “empty” liposome, or in the contra-lateral muscle (uninjected) or the major organs (liver, lung, spleen, or kidney) of mice that were injected with the GNE-lipoplex (Fig. 1a).

**Figure 2:**
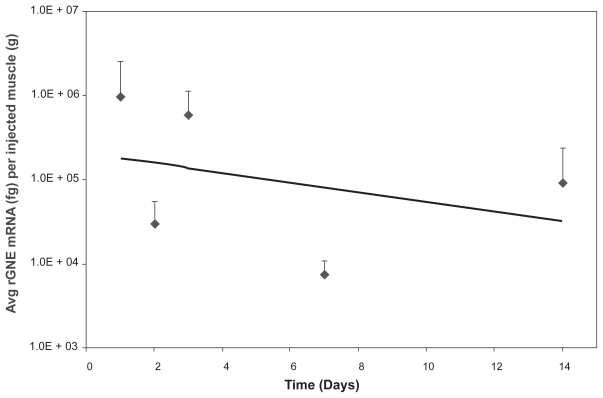
Recombinant human GNE mRNA expression in injected muscles over time. Mice were administered 40 μg GNE-lipoplex, IM and sacrificed at different time points. Tissues (injected muscle, non-injected muscle, and spleen) were harvested and processed for RT-qPCR analysis. Recombinant human GNE mRNA was detected exclusively in the injected muscle. Mean (± SD) rGNE mRNA expression in the injected muscle (n = 3 at each time point). The regression line illustrates a steady decrease of recombinant human GNE mRNA expression over time.

Follow-up analysis using a tracking dye (India Ink) allowed the identification of human rGNE mRNA expression in all the GNE-lipoplex injected animals. Animals were injected with 40 μg GNE-lipoplex mixed with India Ink, and rGNE mRNA expression examined at various times (24 hr, 48 hr, 72 hr, weeks 1 and 2 post IM injection, 3 animals per time point). The injected muscle was identified by its dye uptake from 24 hr up to 2 weeks post-injection. Consistent with the earlier study, rGNE mRNA expression was detected for up to 2 weeks post-injection, and exclusively distributed in 100% of the injected muscle tissue in all animals. There was no rGNE mRNA expression detected in the uninjected muscle or spleen at any time post-injection. Recombinant GNE mRNA expression pattern in the injected muscle was robust at 24 hours post injection (9.3E + 05 fg rGNE mRNA per gram muscle), and detectable levels of GNE were reduced over time (9.0E + 04 fg rGNE mRNA per gram muscle, Fig. 2). Expression levels at 2 weeks post IM injection were comparable between the initial study (4.3E + 04 fg rGNE mRNA per gram of muscle, Fig. 1b) and the follow-up study (Fig. 2), with the slightly lower expression level in the initial study attributed to sampling variance of the injected muscle tissue.

### NOAEL determination for a single IV injection

**Figure 3:**
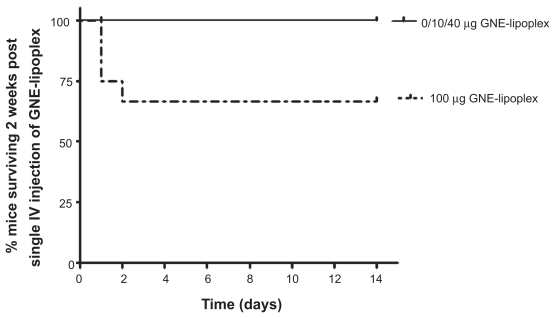
Kaplan-Meier survival analysis for mice administered a single intravenous injection of GNE-lipoplex. Mice were administered a single intravenous injection of 0 μg, 10 μg, 40 μg, or 100 μg GNE-lipoplex. Survival was observed over 2 weeks. Four of 12 mice in 100 μg GNE-lipoplex cohort died within 24 hours after injection. The remaining eight mice in the 100 μg GNE-lipoplex cohort and 2 mice in 40 μg GNE-lipoplex cohort showed acute toxicity (ruffled coat, hunched posture) within 24 hr of IV injection, but recovered within 48 hours and survived for 2 weeks.

In order to determine safety for a single IV injection of GNE-lipoplex, mice were injected either with 10 μg, 40 μg, or 100 μg GNE-lipoplex, and their survival monitored for 2 weeks. Three of 12 mice in 100 μg GNE-lipoplex cohort died within 24 hours after injection and 1 mouse died at 48 hours post-injection (Table 3 and Fig. 3). The remaining eight mice in the 100 μg GNE-lipoplex cohort (2 females, 6 males) and 2 female mice in 40 μg GNE-lipoplex cohort showed acute toxicity (ruffled coat, hunched posture) within 24 hr of IV injection, but recovered within 48 hours. There were no subsequent adverse events noted during the 2-week observation period for the surviving mice in all cohorts (Table 3). Treatment induced weight loss was not evident in injected mice when compared to their baseline weights (mean weight of female mice in grams; pre-treatment: 17.9 ± 1.1, week 1 post treatment: 18.5 ± 1.1, week 2 post-treatment: 19 ± 1.1; mean weight of male mice in grams; pre-treatment: 26.2 ± 1.3, week 1 post treatment: 26.1 ± 1.5, week 2 post-treatment: 26.9 ± 1.6).

Complete blood count analysis did not indicate any significant differences among the various hematology parameters examined in the 4 cohorts at 2 weeks post-injection (Table 3), after exclusion of preparation-related clotted specimens from the various cohorts. There was no remarkable difference in blood chemistry among sera of all 4 cohorts at 2 weeks post-injection (Table 3). Average AST and creatine kinase levels for the 10 μg cohort appeared slightly elevated when compared to the 0 μg cohort, but these values did not reach statistical significance (p = 0.09 for AST assay and p = 0.08 for creatine kinase assay) due to the high standard deviation as a result of a single outlier in the group. AST and creatine kinase levels were not significantly elevated in cohorts that received 40 μg or 100 μg GNE-lipoplex (p > 0.05) (Table 3) suggesting that the elevated values observed for the 10 μg cohort were not due to GNE-lipoplex. Albumin levels were reduced in the cohorts injected with either 40 μg or 100 μg of GNE-lipoplex (3.2 ± 0.2 g/dL). However, these values were within the normal range for the assay (Table 3).

There were no gross pathological changes observed in the liver, kidney, lungs, or spleen harvested from necropsied mice from the 4 cohorts. Histopathological evaluations did not demonstrate inflammatory lesions, or evidence of necrosis. There was also no indication of any severe degenerative change in the tissues. In view of the transient behavioral toxicity observed in mice that received the 40 μg dose, the NOAEL dose was determined to be >10 μg and ≤40 μg GNE-lipoplex for a single IV injection.

### Recombinant human GNE (rGNE) mRNA expression analysis after IV injection

**Figure 4:**
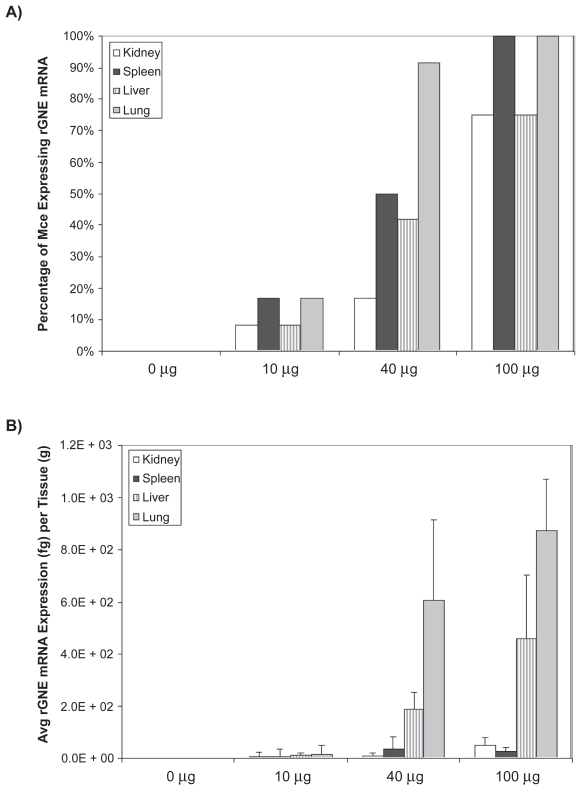
Recombinant human GNE mRNA expression post intravenous injection. Mice were administered a single intravenous injection of 0 μg, 10 μg, 40 μg, or 100 μg GNE-lipoplex. At the end of 2 weeks major organs (kidney, spleen, liver, and lungs) were collected. Total RNA was extracted and real-time qPCR analysis was performed. **A**) The percentage of mice expressing rGNE mRNA per tissue is dose dependant, with at least 75% of the animals expressing rGNE mRNA at the 100 μg dose. **B**) Dose dependant rGNE mRNA expression, predominantly in the lungs and liver.

To characterize human rGNE expression post-intravenous infusion, total RNA was extracted from spleen, lung, liver, and kidney 2 weeks post IV injection. Muscles were not harvested because expression was not expected to be detected beyond 96 hours post injection, based on previous studies.^21^,^22^ Analysis of qPCR data suggest a dose-dependent expression of the GNE-transgene based on the number of mice (n = 12 except for n = 8 for the 100 μg cohort) expressing detectable rGNE mRNA and the amount of rGNE mRNA expressed per tissue (Figs. 4a,b). Recombinant GNE mRNA was not detected in control treatment cohort. At the 100 μg dose, at least 75% of the animals had detectable levels of rGNE in all tissues, with a majority of the expression located in the liver and lung, as expected based on findings from other groups.^23^

## Discussion

The pathologic mechanism for muscle fiber degeneration is not clear. Reduced availability of sialic acid, due to GNE mutations leading to hyposiaylation of muscle proteins is proposed to be a key, if not a sole factor responsible for muscle degeneration in HIBM2.^14^,^17^,^18^ Our current study demonstrates that recombinant human GNE mRNA could be expressed from a wt-GNE plasmid vector *in vivo*. This approach was found to be safe when GNE-lipoplex was administered as a single IM or IV injection up to 40 μg. These findings were supported by 100% survival in IM injected mice and the lack of hematology, blood chemistry, or histopathological abnormalities up to the maximum tested dose of 40 μg. As a result, the NOAEL for IM dosing was considered to be ≥40 μg. In view of the observed transient toxicity at 40 μg dose, the NOAEL for IV administration was considered to be >10 μg and ≤40 μg.

Previous studies have shown that acute toxicity is dose dependant on DNA-lipoplexes.^24^–^26^ These studies demonstrated that naked DNA or empty liposomes had little toxic effect, but the DNA-lipoplex was toxic at high doses. This toxicity was a result of the lipoplex, and not the specific DNA payload. Morbidity within 48 hours was primarily due to severe liver damage, apoptosis of endothelial cells in the lung, and systemic release of proinflammatory cytokines. Intravenous injections containing 100 μg^27^ or >80 μg^28^ DNA-lipoplex lead to acute toxicity, which is in accordance with our IV findings with the 100 μg injected cohort. Although the maximum IM injection volume in humans will vary depending on the muscle size and location, several hospitals follow the guideline of 1cc in the deltoid or 4cc in large muscles, such as the gluteus.^29^ For comparison purposes, an injection of 20 ul (10 ug) in a 20 g mouse is equivalent to 80 mls (40 mg) in an 80 kg human. For clinical trials, initial IM injection volumes would be significantly lower to reduce possible tissue damage due to large volumes of injected material. Results for these studies adequately cover the safe, maximum dose that can be injected into human muscle.

Although the target tissue for gene therapy of HIBM2 is skeletal muscle, there is significant muscle loss in patients with the disease.^30^ The limited mass of the affected muscle makes IM injections difficult, due to the limited volume that can be administered. An intravenous infusion of the GNE-lipoplex would address the issue of not having enough tissue mass for injections. Previous studies by Templeton et al. have demonstrated distribution of DNA-lipoplexes (similar to the ones used for the current animal study), to all major organs, including skeletal muscles.^31^ In those studies, lung was the primary target tissue, with skeletal muscle demonstrating approximately 1.5 logs less reporter gene expression. Our study was comprised of a single IM or IV injection to demonstrate the effectiveness of our GNE-lipoplex delivery and safety. Studies are currently underway to detect the trafficking of sialic acid to determine if organs such as the liver can excrete labeled sialic acid for uptake in distant organs such as skeletal muscle. Future studies, including administration of GNE-lipoplexes via multiple injections are being planned to determine expression profile of the GNE transgene and observe any toxicity due to re-administration of the lipoplex.

Attempts to produce mouse models to study the pathophysiological mechanism of the disease have proved to be difficult. A limb girdle muscular dystrophy hamster model was generated and 97% of the animals were rescued in muscle strength following a single IM injection with an adeno-associated virus (AAV) containing δ-sarcoglycan (SG) gene.^32^ However, generation of GNE deficient mice by inactivation of UDP-GlcNAc 2-epimerase led to embryonic lethality.^33^ Although heterozygous GNE deficient mice were viable and demonstrated 25% reduction in membrane bound sialic acid, these mice did not develop myopathy even after 2 years.^34^ Generation of knock-in mice harboring the M712T GNE/MNK mutation led to the development of homozygous mutant mice pups that did not survive beyond the 3rd day after birth and demonstrated renal abnormalities and podocytopathy 2 days after birth.^35^ The recent report by Malicdan et al. (36) created transgenic GNE^(−/−)^ mice expressing the human GNE mutation D176V. These mice exhibited some similar biochemical defects as afflicted patients (hyposialyation, beta-amyloid deposition, rimmed vacuole formation) at 32 weeks, but the pathological features of this transgenic model (involvement of involuntary muscles, high expression levels of the mutant GNE gene in skeletal muscles) were not consistent with the human pathology. Thus this animal model has limited applicability for the study of experimental therapy of HIBM. In spite of absence of a relevant animal model, the specific detection of human GNE mRNA in mouse tissues after a single IM injection is a potentially promising first step towards HIBM2 gene therapy.

Prior studies have shown that GNE mutation probably affects the functional status, and not total protein expression of the GNE gene.^37^ HIBM2 patients and normal control subjects demonstrated similar levels of GNE expression by western blot and immunohistochemistry, although neither technique can discriminate between wild type vs. mutant GNE. In our studies GNE expression was analyzed using real-time qPCR, which is highly sensitive and specific for the human wild type recombinant GNE gene. Using this technique we were able to detect up to femtograms of the rGNE mRNA in the mouse tissues, based on specific primers that uniquely amplify the human GNE transcript (recombinant GNE mRNA transgene). Functional assays are currently under development to confirm enhanced enzymatic activity.

AAV viruses have been used for IV injection to deliver transgenic DNA systemically to most skeletal muscles in neonatal dogs.^38^ Unfortunately, most viral vectors can not be injected repeatedly due to the immunogenicity of the virus. Cationic DOTAP:Cholesterol liposomes are the most commonly used liposomes for gene delivery in various research and clinical studies. One of the advantages of using these liposomes is that their low immunogenicity allows for repeated injections to achieve maximum and long term expression *in vivo*.^39^ Mouse muscle tissue was the third highest tissue in terms of CAT reporter gene expression when mice were injected intravenously with DOTAP:Chol complexed with the CAT reporter plasmid.^31^ This formulation of liposomes has been shown to penetrate several tight layers of smooth muscle cells in the arteries of pigs^40^ and has been used for gene delivery of various tumor suppressor genes with evidence of effectiveness in treatment of primary and disseminated lung cancers.^41^,^42^

There are currently no effective treatments for HIBM2 patients. In our studies, we have demonstrated reconstitution of enzymatically active protein *in vitro*^19^ and recombinant GNE mRNA expression in muscles injected with 10 or 40 μg GNE-lipoplex. However, we were not able to demonstrate sialic acid reconstitution in this study because of the presence of endogenous, wild type activity in the experimental host. Functionally, the wild type GNE enzyme is autoregulated by its downstream product, CMP-sialic acid.^43^ Therefore, over-expression of the recombinant GNE transgene in the mouse tissues would not correspond to an over-expression of sialic acid. New animal studies are currently under way utilizing the M712T knock-in mice mentioned above. These animals will be injected with GNE-Lipoplex and monitored to determine if the animals survive beyond day 3 after birth. This animal model will not immediately address the efficacy of the wild type GNE for rescued muscle function, but will assess the safety of the GNE-Lipoplex and ability of the transgene to rescue the animal from the knock-in mutation.

Since reconstitution with sialic acid or Man-NAc increased sialyation of proteins in various studies,^33^,^35^,^44^ actually replacing the defective gene in human muscle with its wild type counterpart is hypothesized to be beneficial to patients. This is anticipated to improve patient’s muscle function primarily as a result of resialyation of muscle proteins but other GNE-mediated mechanisms may contribute.

## Figures and Tables

**Table 1:** Study design.

Study number	Injection route	GNE-Lipoplex dose (total volume injected)	Treatment	Mice/cohort	Assessment
I	Intramuscular	10 μg (80 μl)	GNE-lipoplex	6 female, 6 male	Safety and toxicology,
		40 μg (80 μl)	GNE-lipoplex	6 female, 6 male	GNE expression
		0 μg (80 μl)	Empty liposomes	6 female, 6 male	
		No injection	–	6 female, 6 male	
II	Intramuscular	40 μg (80 μl)	GNE-lipoplex	15 female	GNE expression
		0 μg (80 μl)	Empty liposomes	6 female	
III	Intravenous	10 μg (200 μl)	GNE-lipoplex	6 female, 6 male	Safety and toxicology,
		40 μg (200 μl)	GNE-lipoplex	6 female, 6 male	GNE expression
		100 μg (200 μl)	GNE-lipoplex	6 female, 6 male	
		0 μg (200 μl)	Empty liposomes	6 female, 6 male	

**Table 2:** Toxicology assessments of mice injected with a single, intramuscular injection of GNE-lipoplex.

	Uninjected (n = 12)	0 μg (n = 12)	10 μg (n = 12)	40 μg (n = 12)
Survival	12/12	12/12	12/12	12/12
Treatment induced body weight decrease	0/12	0/12	0/12	0/12
Acute toxicity (ruffled coat, hunched posture), mice recovered in 48 hours after injection	0/12	0/12	0/12	0/12
Lesions at necropsy	0/12	0/12	0/12	0/12
Lesions observed by histopathology caused by treatment	0/12	0/12	0/12	0/12
**Bone marrow function**	**Uninjected (n = 4)**	**0 μg (n = 5)**	**10 μg (n = 2)**	**40 μg (n = 5)**
WBC (×10^3^/μL)	3.44 ± 1.47	2.58 ± 0.88	2.35	4.38 ± 1.5
RBC (×10^6^/μL)	8.44 ± 0.65	7.79 ± 0.73	8.81	8.43 ± 0.75
Platelets (×10^3^/μL)	520.75 ± 374.78	396.2 ± 343.05	569.50	428.2 ± 346.31
**Serum chemistry**	**Uninjected (n = 6)**	**0 μg (n = 6)**	**10 μg (n = 6)**	**40 μg (n = 6)**
AST (U/L)	172 ± 42	305 ± 65*	323 ± 271	141 ± 88
ALT (U/L)	50 ± 11	61 ± 12	58 ± 18	41 ± 17
Total bilirubin (mg/dL)	0.1	0.1	0.1	0.1
Alkaline phosphatase (U/L)	94 ± 17	95 ± 17	97 ± 13	96 ± 18
Creatine kinase (U/L)	1052 ± 290	2110 ± 571*	2349 ± 2507	948 ± 851
BUN (mg/dL)	20 ± 4	22 ± 3	24 ± 9	17 ± 4
Albumin (g/dL)	3.1 ± 0.2	3.1 ± 0.2	3.4 ± 0.2	3.1 ± 0.2

* p< 0.05 compared to uninjected cohort.

**Table 3:** Toxicology assessments of mice injected with a single, intravenous injection of GNE-lipoplex.

	0 μg (n = 12)	10 μg (n = 12)	40 μg (n = 12)	100 μg (n = 12)
Survival	12/12	12/12	12/12	8/12
Treatment induced body weight decrease	0/12	0/12	0/12	0/12
Acute toxicity (ruffled coat, hunched posture), mice recovered in 48 hours after injection	0/12	0/12	2/12	8/12
Lesions at necropsy	0/12	0/12	0/12	0/12
Lesions observed by histopathology caused by treatment	0/12	0/12	0/12	0/12
**Bone marrow function**	**0 μg (n = 6)**	**10 μg (n = 4)**	**40 μg (n = 4)**	**100 μg (n = 3)**
WBC (×10^3^/μL)	4.13 ± 0.65	5.2 ± 0.73	4.56 ± 1.09	2.7 ± 0.1
RBC (×10^6^/μL)	8.65 ± 0.65	8.61 ± 0.76	8.92 ± 0.11	8.48 ± 0.45
Platelets (×10^3^/μL)	623.33 ± 430.04	970 ± 378	882.75 ± 270.11	792 ± 374.2
**Serum chemistry**	**0 μg (n = 6)**	**10 μg (n = 6)**	**40 μg (n = 6)**	**100 μg (n = 4)**
AST (U/L)	102 ± 19	144 ± 52	119 ± 28	88 ± 30
ALT (U/L)	48 ± 11	45 ± 12	39 ± 14	39 ± 4
Total bilirubin (mg/dL)	0.1	0.1	0.1	0.1
Alkaline phosphatase (U/L)	101 ± 14	104 ± 24	100 ± 22	101 ± 19
Creatine kinase (U/L)	455 ± 122	776 ± 390	542 ± 131	387 ± 220
BUN (mg/dL)	20 ± 5	19 ± 4	17 ± 3	15 ± 3
Albumin (g/dL)	3.5 ± 0.2	3.3 ± 0.3	3.2 ± 0.2	3.2 ± 0.2
